# Meningoencephalitis due to the amoeboflagellate *Naegleria fowleri* in ruminants in Algeria

**DOI:** 10.1051/parasite/2016011

**Published:** 2016-03-15

**Authors:** Mohamed Seghir Benterki, Ammar Ayachi, Omar Bennoune, Estelle Régoudis, Michel Pélandakis

**Affiliations:** 1 Veterinary Clinic “Le refuge” 05000 Batna Algeria; 2 Laboratory ESPA, University of Batna 1 05000 Batna Algeria; 3 Université de Lyon, UMR 5240, Université Claude Bernard Lyon1 69622 Villeurbanne France

**Keywords:** Meningoencephalitis, Naegleria fowleri, Free-living amoeba, Real-time PCR, Diagnosis, Cattle

## Abstract

Primary amoebic meningoencephalitis (PAM) is a fatal infection in most cases, caused by the amoeba flagellate *Naegleria fowleri*. This report describes the first cases of PAM in Algeria, in a cow and a ewe from Batna, north-eastern Algeria. The death of both ruminants occurred a week after the first clinical manifestations. The cerebrospinal fluid, after staining with May-Grünwald-Giemsa, showed the presence of amoebae cells. Histological sections revealed numerous amoebae in all parts of the brain. The presence of *N. fowleri* was confirmed using a species-specific real-time PCR in histological tissue sections. The two PAM cases were reported during the hot season, and the source of infection is very likely the water where the cattle came to drink. Particular attention should be focused on this type of infection in aquatic environments when the temperature is high and preventive measures must be taken to avoid the proliferation of *N*. *fowleri*.

## Abstract (fr)

La méningo-encéphalite amibienne primitive (MAP) est une infection mortelle dans la plupart des cas, causée par l’amibe flagellée *Naegleria fowleri*. Ce rapport décrit les premiers cas de MAP en Algérie, chez une vache et une brebis à Batna, au nord-est du pays. La mort des deux ruminants s’est produite une semaine après les premières manifestations cliniques. L’examen du liquide céphalorachidien après coloration au May-Grünwald-Giemsa a montré la présence de cellules amibiennes. Des coupes histologiques ont révélé la présence de nombreuses amibes dans toutes les parties du cerveau. La présence de *N. fowleri* a été confirmée par PCR spécifique en temps réel à partir des coupes histologiques. Les deux cas de MAP ont été signalés au cours de la saison chaude, et la source d’infection est vraisemblablement l’eau où le bétail venait boire. Une attention particulière devrait être accordée à ce type d’infection dans les milieux aquatiques lorsque la température est élevée et des mesures préventives doivent être prises pour éviter la prolifération de *N. fowleri*.

## Introduction

Most cases of meningoencephalitis in animals are due to viral and bacterial pathogens. Some protists however are causative agents, and particularly the amoeboflagellate *N. fowleri* responsible for meningoencephalitis, which is called primary amoebic meningoencephalitis (PAM) [1, 3]. This fast and fatal infection is usually observed in healthy young people exposed to warm water. The infection occurs by inhalation of contaminated water with *N. fowleri* which passes through the nasal mucosa, then the olfactory nerves and migrates to the brain [7]. PAM can also affect animals and has been experimentally induced in mice, sheep and monkeys [15–17]. However, there are few naturally acquired cases of PAM described in animals. Four cases occurred naturally in cattle from California, Costa Rica and Brazil [2, 8, 10, 14] but these were not clinically diagnosed in living animals. An additional case was described in a captive South American tapir [6].

In the present study, we report two cases of PAM which were observed in a cow and a ewe from Algeria. All clinical examinations were carried out on the animals before death. Molecular analysis, which was carried out subsequently in France, allowed us to identify *N. fowleri* as the agent responsible for this infection. A calf and two ewes living in the same area have previously shown similar symptoms, suggesting they were also most probably infected with *N. fowleri*.

## Case report

Near Batna at an altitude of 1200 m, in north-eastern Algeria, a 4.5-year-old, 7-month pregnant Simmental cow, vaccinated against rabies and foot and mouth disease, lying down for two days showed the following symptoms: fever, anorexia, tiredness, constipation and hyporeactive nervous disorders. The following examinations were carried out at the Institute of Veterinary and Agricultural Sciences of the University of Batna. A lumbar puncture showed cloudy and haemorrhagic cerebrospinal fluid (CSF) with a low concentration of glucose and a high concentration of protein, suggesting meningoencephalitis. Bacterial meningitis was firstly suspected and an appropriate treatment (Ampicillin + TMP-Sulfa + thiamine) was given without remission. At day 5, a blood analysis showed the following values: WBC = 16,000, urea = 1.50 g/L, creatinine = 32 mg/L, Na+ = 140 meq/L, K+ = 4.2 meq/L, Cl− = 110 meq/L, TGO = 1200 UI/L, TGP = 70 UI/L, CK = 940 UI/L, LDH = 940 UI/L, PT = 62 g/L and ALB = 37 g/L. The cow fell into a coma and died 7 days after the first clinical manifestations. Blood analysis revealed severe kidney and heart complications that led to death.

Further analysis of fresh CSF, which was also performed in Batna, indicated glucose at a concentration of 30 mg/dL, proteins at a concentration of 47 mg/dL and a cell count of 160 cells/mm^3^. Blood May-Grünwald-Giemsa stained smears showed 75% polymorphonuclear (PMN), 25% lymphocytes and no anaemia. No bacteria or fungus was found in the fresh CSF. However, several cells appeared with approximately 20–25 μm size with pseudopodia, suggesting the presence of amoeba trophozoites. The eruptive movement of pseudopodia as well as the cell morphology suggested the genus *Naegleria*. CSF smears stained with methylene blue after 24 h showed round cells (10 μm) with a vacuolated cytoplasm.

The trophozoite cells from CSF were cultured on non-nutrient agar overlaid with a lawn culture of *Escherichia coli* as a food source. The amoeba trophozoites were observed after 3 days of incubation at 37 °C and increased in number until 6–7 days. The cystic forms appeared for 14 days. A flagellation test was performed to confirm the presence of *Naegleria*. In contrast to other pathogenic amoebae, such as *Acanthamoeba* and *Balamuthia*, *Naegleria* was able to flagellate at 37 °C in hypotonic medium (distilled water). The flagellation test was positive with cells harbouring two flagella and the transformation from trophozoites to flagellates occurred within 48 h.

One week after the case of the cow, a 5-year-old pregnant ewe in the last third of gestation fell ill with the same symptoms and died 6 days later. Clinical examinations gave similar results to those obtained with the cow, with the presence of amoeba trophozoites in the CSF.

Careful examination after necropsy and the opening of the cranial cavity revealed a scanty black fluid, which flowed from sinuses and congested meninges. For histopathological studies, small samples were excised from the frontal cortex of the cow and ewe brains, fixed in formalin 10% then dehydrated and embedded in paraffin wax and later stained with haematoxylin and eosin followed by careful microscopic examination. The histological sections (*n* = 6) from the frontal cortex showed trophozoite cells in the two samples.

### Examination in France

The fresh isolate could not be maintained in culture in Batna and was lost. Only histological tissue sections fixed in 10% formalin were available to carry out a molecular diagnosis. Consequently, genomic DNA was extracted from formalin-fixed brain tissue sections using the UNSET method [4] and sent to the French Laboratory.

**Figure 1.**
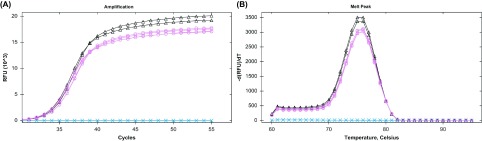
Specific detection of *N. fowleri* by real-time PCR. The DNA was extracted from formalin-fixed brain samples which were stored for 17 months. (A) Real-time PCR amplification curves of DNA from cow and ewe samples demonstrating the presence of *N. fowleri*. (B) The corresponding melting curve profiles which were equivalent to that of *N. fowleri*. (∆) cow sample; (o) ewe sample; (x) negative control.

A real-time PCR specific to *N. fowleri* was performed a few months later from histological tissue sections fixed in 10% formalin. The real-time PCR was performed using specific primers, the upper primer Fow-A1, 5′-TTCCGAACCCACTCAATAAA-3′ and the lower primer Fow-A2, 5′-TTGGCAATCGTGAAGTAAAC-3′, which were used to target a 118-bp fragment [9, 11]. The real-time PCR was carried out on a CFX96 real-time thermal cycler (Bio-Rad Laboratories, Hercules, CA) by using SYBR green. Each reaction mix contained 5 μL of LightCycler 480 SYBR Green I Master (Roche Life Science), 1 μL of primers (10 μM), 2 μL of target DNA and 1.5 μL of sterile water, with a final reaction volume of 9.5 μL. The PCR cycling programme was as follows: 95 °C for 5 min., followed by 5 cycles at 95 °C for 10 s, 65 °C for 20 s, and 72 °C for 20 s, and 55 cycles at 95 °C for 10 s, 58 °C for 20 s, and 72 °C for 20 s. The melting curve was obtained by a gradual increase of temperature from 60 °C to 95 °C incremented by 1 °C/5 s. No-template and negative amplification controls were also included with each run. The specific PCR results revealed a positive signal for the cow and ewe with an identical melting temperature of the amplicons corresponding to that of *N. fowleri* (Fig. 1). The PCR products were also sequenced and were identical to the *N. fowleri* sequence.

## Discussion

The two PAM cases were reported during the July heat waves. It is very likely that the source of infection was the water where the cattle came to drink. The drinking water came from a deep well flowing into an irrigation basin of small capacity exposed to an ambient temperature that approximates 45 °C. During this period, the drinking trough contained small amounts of hot water where the temperature was higher than 30 °C. Such a situation was suitable for the proliferation of the thermophilic species, *N. fowleri*, which can tolerate temperatures higher than 45 °C [12, 15].

Morphological analyses and flagellation tests after cultivation allowed determination of the genus as *Naegleria*, but this was not sufficient to identify the pathogenic species [13]. Unfortunately, for further molecular analyses the pathogenic strain could not be maintained in culture and was lost. A formalin-fixed tissue section was the only material available for extracting DNA in order to unambiguously identify the pathogenic agent responsible for the meningitis. PCR did not always give satisfactory results because formalin fixation for sample conservation is detrimental to DNA quality [5]. On the other hand, a real-time PCR-based method that we have developed provided diagnostic results from brain samples which were stored for 17 months in 10% formalin (in the absence of phosphate-buffered saline (PBS)). Regrettably, water samples could not be taken from the sites since the site of infection, the water basin, was decontaminated shortly after the discovery of meningitis and the local authorities closed and destroyed the basin and drilling.

No human cases of PAM have been reported in Algeria or the Maghreb. The same is true for animals for which ruminant meningitis was attributable to viral infectious diseases.

Here we report the first cases of PAM in Algeria. These two cases may not be isolated, since other cows and ewes exhibited the same symptoms a few weeks before.

A significant number of meningitis cases are now reported in cattle in various geographic locations worldwide, suggesting that meningitis caused by the genus *Naegleria* is not so rare in cattle. PAM is certainly underdiagnosed since clinical symptoms are very similar to those of other more commonly known types of meningitis [15]. Therefore, particular attention should be focused on this type of infection. In this case, the circumstances under which the infection appeared were an important indication for establishing the diagnosis. The thermophilic *N. fowleri* occurs worldwide in soil and aquatic environments, and tends to proliferate in the hot season. Therefore, drastic preventive measures must be taken and stagnant water in warm areas monitored appropriately. Similarly, it is important that clinicians and biologists pay special attention to the direct examination of CSF following meningitis.
